# Supplementary material for: Global syndromes induced by changes in solutes of the world’s large rivers
Source: Nat Commun. 2021 Oct 12;12:5940. doi: 10.1038/s41467-021-26231-w (PMC8511150; doi:10.1038/s41467-021-26231-w)
Supplement: Supplementary file 1 — Supplementary Information [file 41467_2021_26231_MOESM1_ESM.pdf]

Supplementary Information for

Global syndromes induced by changes in solutes of the world's large rivers

Jiang Wu[1,2,8], Nan Xu[2,8], Yichu Wang[3], Wei Zhang[4], Alistair G.L. Borthwick[5,6], Jinren Ni[1,7*]

[1]State Environmental Protection Key Laboratory of All Materials Fluxes in River Ecosystems, Peking University, Beijing 100871, P. R. China;

[2]The Key Laboratory for Heavy Metal Pollution Control and Reutilization, School of Environment and Energy, Peking University Shenzhen Graduate School, Shenzhen 518055, P. R. China;

[3]College of Water Sciences, Beijing Normal University, Beijing 100875, P. R. China;

[4]Department of Plant, Soil and Microbial Sciences, Michigan State University, East Lansing, MI 48824, United States;

[5]Institute of Infrastructure and Environment, School of Engineering, The University of Edinburgh, The King's Buildings, Edinburgh EH9 3JL, UK;

[6]School of Engineering, Computing and Mathematics, University of Plymouth, Drake Circus 14 Plymouth, PL4 8AA, UK;

[7]Center for Global Large Rivers, School of Environmental Science and Engineering, Southern University of Science and Technology, Shenzhen 518055, P. R. China.

[8]These authors contributed equally: Jiang Wu, Nan Xu

*Correspondence and requests for materials should be addressed to J.R.N. (jinrenni@pku.edu.cn).

This PDF file includes:

Supplementary Methods

Supplementary Figs. 1 to 10

Supplementary Tables 1 to 9

Supplementary References 1 to 87

Supplementary Methods

1. Database compilation

To construct a global database of major dissolved ions in the world's large rivers (≥ 1,000 km^2)[1], we compiled information from 12 publicly available databases (listed in Supplementary Table 7) and also extracted long-term hydrochemical data from the open literature[2-37].

We consolidated these data in a global dataset of major dissolved ions and total dissolved solids (TDS) in the world's large rivers. First, we combined all the data from all our sources. Then, for any overlapping data at any given station in any given year, if the difference was less than 10%, we calculated the average of all reported data from all the databases. If the discrepancy was greater than 10%, we used expert judgement and usually preserved the value from whichever record was the longer. Annual average concentrations of 8 major dissolved ions (Ca^{2+}, Mg^{2+}, Na^{+}, K^{+}, SO_4^{2-}, Cl^{-}, HCO_3^{-} and dissolved silica (DSi)) were calculated by averaging measured values at finer time steps (monthly or seasonally) whenever available. Otherwise, average annual concentrations were taken from values reported in the literature. In cases where TDS data were already available in the data sources, we used these records. In cases of any missing TDS measurements, we simply summed the concentrations of all DS (Ca^{2+}, Mg^{2+}, Na^{+}, K^{+}, SO_4^{2-}, Cl^{-}, HCO_3^{-} and DSi) to determine TDS when all the DS values were available. For the TDS data, we obtained 8,016 data points directly from open databases, 485 data points from model results (explained in more detail in Section 2, Supplementary Methods), 121 data points from the literature, and 543 data points by summing the

concentrations of all dissolved ions. Thus, about 87% of the TDS data were taken directly from public databases.

In addition to the global database of TDS, we also compiled the annual average flows of the world's large rivers in the PKU-IEE-WLRs-WS-NL2006 Database. Supplementary Table 8 lists the source databases for the annual river discharge data. Any missing data were filled by river discharge data taken from the literature and online sources[2-37]. We consolidated all the river discharge data using an approach similar to that for the dissolved solids database, particularly overlapping data.

In summary, our database includes data from 600 stations in 149 rivers located across six continents, as shown in Fig. 1 of the main text. The stations are distributed as follows: 54.8% in North America, 14.8% in Asia, 12.2% in Oceania, 8.2% in Europe, 7.5% in South America, and 2.5% in Africa. The percentage of stations with records no less than 5 years ranged between 82-86% for all DS. Moreover, 43–52% of stations had records of duration 10-30 years for 8 major dissolved ions and TDS (Supplementary Table 9). To our knowledge, this database is the most comprehensive to date in terms of spatial and temporal coverage of DS.

2. Data quality

2.1 Outlier detection

To control data quality (and ensure robust trend analysis and flux calculations), we removed outliers in the database, as is usual practice[38]. First, we tested whether the time series data for any of DS at any given station followed a normal distribution. If so, then outliers were identified using the Grubbs' test[39] using the R package 'outliers'. If not,

any data points beyond three standard deviations (SD) of the mean[40] were considered outliers. We removed 1,273 outliers using the Grubbs' test and 615 outliers using the three SD method. The percentage number of removed outliers was 2.3% of the total data points.

2.2 Charge balance

We selected 6,124 annual average concentration data samples containing all major charged ions ($Ca^{2+}/Mg^{2+}/Na^{+}/K^{+}/SO_4^{2-}/Cl^{-}/HCO_3^{-}$) to assess data quality using the charge balance approach (in meq = 10^{-3} equivalents per liter). This method compared the total charge carried by total dissolved cations ($TZ^{+} = Na^{+} + K^{+} + 2Mg^{2+} + 2Ca^{2+}$) to that by total dissolved anions ($TZ^{-} = Cl^{-} + HCO_3^{-} + 2SO_4^{2-}$). Given that all dissolved ions in water should retain total neutrality, the normalized inorganic charge balance ($NICB = (TZ^{+} - TZ^{-})/(TZ^{+} + TZ^{-}) \times 100\%$)[31, 41] should be less than 10% on average. In our database, 5,447 annual average data (89%) met this criterion, indicating high data quality (within the usual combined analytical uncertainty for all measurements[6, 42]).

3. Flux calculation

To calculate the annual fluxes of DS in the world's large rivers, we used a hybrid approach that utilized observed annual concentrations and river flows for direct calculation supplemented with LOAD ESTimator model results for any missing data. The direct calculation and modeling methods are described below.

3.1 Direct calculation

Solute flux was calculated as the product of annual average concentrations (C_i) and

annual average flow (Q_i) as follows:

$$F_{\mathrm{ea}} = k \times C_i \times Q_i \qquad \text{(S1)}$$

where $k = 0.001$ is the conversion coefficient, F_{ea} is the annual flux, C_i is the annual average concentration of Ca^{2+}, Mg^{2+}, Na^{+}, K^{+}, SO_4^{2-}, Cl^{-}, HCO_3^{-}, and DSi, and Q_i is the annual average flow for a given year at a specific station.

3.2 LOADEST Model

For any undocumented (or missing) data, we used the LOAD ESTimator (LOADEST), a FORTRAN program for estimating constituent loads in streams and rivers based on daily runoff data and calibrated concentrations[43], to estimate any missing annual flux values the direct method could not calculate. Adjusted Maximum Likelihood Estimation (AMLE) was used to fit the calibration equation. Then the regression model with the lowest Akaike Information Criterion (AIC) value was selected as best fit from a nested series of potential models[44]. Finally, we supplemented 4,967 modeled annual average concentrations in our database, accounting for about 6.3% of the entire concentration dataset.

Moreover, we compared modeled with observed annual average concentrations and fluxes (Supplementary Fig. 10) for 15,702 data points. Whereas the modeled concentrations were on average 21% lower than the observed concentrations (Supplementary Fig. 10a), the modeled and observed fluxes were similar with a regression slope of 0.97 ($R^2 = 0.96$). This confirmed that the modeling approach was able to estimate annual fluxes of dissolved solids in close agreement with direct measurements, demonstrating the validity of our hybrid approach.

3.3 Global fluxes to the oceans

After determining the annual fluxes of DS for all the river stations, we calculated the global fluxes of dissolved solids to the oceans using COSCAT (COastal Segmentation and its related CATchment)[45] which is a well-established tool for estimating nitrogen yield[45], natural riverine silica inputs[46] and river discharge[45, 47] to the oceans. Here, we applied the COSCAT methodology to estimate the fluxes of TDS and major ions (Ca^{2+}, Mg^{2+}, Na^{+}, K^{+}, SO_4^{2-}, Cl^{-}, HCO_3^{-} and DSi) to coastal zones based on our dataset of calculated fluxes with missing information taken from ancillary multi-averaged ion concentrations provided by Meybeck and Ragu[48]. For each COSCAT[47], the fluxes to the oceans were calculated as follows:

For areas where data were available in our database, we computed the average yield Y_j (Mt/(km^2·yr)) in each COSCAT catchment (j = 1 to 151) from

$$Y_j = \frac{\sum_{i=1}^{n} \frac{F_{\text{basin } ij}}{A_{\text{basin } ij}}}{n_j} \quad \text{(S2)}$$

and the fluxes in each COSCAT, $F_{\text{COSCAT } j}$ (Mt/yr) from

$$F_{\text{COSCAT } j} = Y_j \times A_{\text{COSCAT } j} \quad \text{(S3)}$$

where $F_{\text{basin } ij}$ is an individual flux in the i-th sub-basin of the j-th COSCAT catchment for stations in our database (Mt/yr), A_{basin} is the area of each sub-basin (km^2), n is the number of sub-basins, and $A_{\text{COSCAT } j}$ is the area of each COSCAT (km^2) taken from Meybeck et al.[45].

For the remaining COSCATs where data were not available in our database and the dataset in Meybeck and Ragu[48] was instead used, we calculated the discharge-weighted ion concentration Ion_{*j} (mg/L) from

$$\text{Ion} *_j = \frac{\sum_{i=1}^{n}(C_{ij} \times Q_{ij})}{\sum Q_j} \quad \text{(S4)}$$

and $F_{\text{COSCAT}\,j}$ in each COSCAT (Mt/yr) from

$$F_{\text{COSCAT}j} = k \times \text{Ion} *_j \times Q_{\text{COSCAT}j} \quad \text{(S5)}$$

where $k = 0.001$ is the conversion coefficient, C_{ij} and Q_{ij} are the concentrations (mg/L) and corresponding river discharge (km^3/yr) at the i-th station of the j-th COSCAT catchment using information from the dataset of Meybeck and Ragu[48], Q_{COSCAT} (km^3/yr) was extracted from Durr et al.[46], and n is the number of stations in each COSCAT area.

For areas without any documented data, we calculated the fluxes in each COSCAT according to the method proposed by Durr et al.[46]. We first calculated the discharge-weighted ion concentrations Ion_{**j} (mg/L) in adjacent documented areas (using either our database or the dataset in Meybeck and Ragu[48]) with similar weathering conditions and catchment properties, from:

$$\text{Ion} **_j = \frac{\sum_{i=1}^{n}(C*_{ij} \times Q*_{ij})}{\sum Q*_j} \quad \text{(S6)}$$

where $C*_{ij}$ and $Q*_{ij}$ are the concentrations and corresponding river discharge at the i-th station in the j-th COSCAT catchment. We then extrapolated the Ion* and Ion** to the undocumented COSCAT catchment[46] and the flux was calculated as:

$$F_{\text{COSCAT}j} = k \times (\text{Ion} *_j \text{ or Ion} **_j) \times Q_{\text{COSCAT}j} \quad \text{(S7)}$$

Finally, the total global fluxes were calculated as the sum of the fluxes in 151 catchments in all COSCAT exorheic areas.

$$\text{Flux} = \sum_{j=1}^{151} F_{\text{COSCAT}j} \quad \text{(S8)}$$

4. Trend analysis

We used non-parametric Mann-Kendall (MK) method[49, 50] to perform trend analysis

of time series of duration ⩾ 5 years. In addition, trend-free pre-whitening (TFPW) in the R package 'modifiedmk' ensured data independence before implementing the MK test[51]. The overall procedure is called TFPW-MK (Trend-Free Pre-Whitening Mann-Kendall).

The MK statistic parameter (S) is determined from

$$S = \sum_{k=1}^{n-1} \sum_{j=k+1}^{n} \operatorname{sgn}(x_j - x_k) \quad \text{(S9)}$$

in which

$$\operatorname{sgn}(x_j - x_k) = \begin{cases} 1,\ x_j - x_k > 0 \\ 0,\ x_j - x_k = 0 \\ -1, x_j - x_k < 0 \end{cases} \quad \text{(S10)}$$

where n is the number of data points in the time series, x_j and x_k are the j-th and k-th values in the new ordered data series obtained after eliminating the autocorrelation of the original data ($j > k$). For $n \geq 10$, the variance of S is obtained, using the normal approximation test, from

$$\operatorname{Var}(S) = \frac{\left[n(n-1)(2n+5) - \sum_{p=1}^{q} t_p(t_p-1)(2t_p+5)\right]}{18} \quad \text{(S11)}$$

where q is the number of tied groups (or groups with the same value), and t_p is the number of samples in the p-th tied group. The Z parameter of the statistical test is:

$$Z = \begin{cases} \frac{S-1}{\sqrt{\operatorname{Var}(S)}}, S > 0 \\ 0\ ,\ \ S = 0 \\ \frac{S+1}{\sqrt{\operatorname{Var}(S)}}, S < 0 \end{cases} \quad \text{(S12)}$$

The time series exhibit an increasing trend with a positive Z value, and a decreasing trend with a negative Z value. The null hypothesis (H_0), which indicates no trend, is true if $-Z_{1-\alpha/2} \leq Z \leq Z_{1-\alpha/2}$ for a two-tailed test, in which α is the significance level. For $n \leq 10$, if S is positive (or negative) and the probability value according to n and S

(or absolute S) is less than $\alpha/2$ for a two-tailed test, then H_0 is rejected, and the trend is either increasing or decreasing. In this study, a confidence level of 95% (α = 0.05) was used. $Z_{1-\alpha/2}$ is the critical value of Z from the standard normal table; for the 5% significant level the value of $Z_{1-\alpha/2}$ is 1.96.

5. Method for determining solute-induced river syndromes

Based on the sum of total cation concentrations (Σ^+, meq/L), Meybeck[52] proposed that 'extremely dilute' rivers with the least mineralized water have $\Sigma^+ < 0.185$ meq/L, 'dilute' rivers have $\Sigma^+ < 0.75$ meq/L, 'medium dilute' rivers have $\Sigma^+ < 1.5$ meq/L, 'medium mineralized' rivers have 1.5 meq/L < Σ^+ < 3 meq/L, 'mineralized' rivers have $\Sigma^+ > 3$ meq/L, and 'saline' rivers with the most mineralized water have $\Sigma^+ > 24$ meq/L. In combination with the trend analysis, we further defined three solute-induced river syndromes: salinization with $\Sigma^+ > 24$ meq/L and an increasing trend; mineralization with $\Sigma^+ > 3$ meq/L and an increasing trend; and desalinization with $\Sigma^+ < 1.5$ meq/L and a decreasing trend.

To determine whether a river experiences acidification or alkalization, both pH and alkalinity (or acidity) must be considered[4, 6, 53]. We calculated the ratio of hardness to alkalinity, and extracted mean pH values for rivers from the GEMS (Global Environment Monitoring System for Water) database[54]. When the ratio of hardness to alkalinity > 1, acid input from anthropogenic sources is likely to be responsible[38]. We therefore propose the following diagnosis procedure. If the water pH < 7 and the ratio of hardness to alkalinity > 1 and increasing with time in a river, the river is experiencing an acidification syndrome. If pH > 7 and the ratio of hardness to alkalinity < 1 and

decreasing with time, the river is undergoing an alkalization syndrome.

Finally, we examined hardness (expressed as calcium carbonate equivalent in mg/L, $CaCO_3$ mg/L) following the WHO classification of water hardness into soft (< 60 mg/L), moderately hard (60-120 mg/L), hard (120-180 mg/L) and very hard (>180 mg/L)[55] levels. Thus, if river water has hardness > 120 mg/L with an increasing trend, the river has a hardening syndrome. Conversely, if river water has hardness < 60 mg/L with a decreasing trend, it has a softening syndrome.

6. Environmental factors extraction and latitudinal distribution

First, the sub-basin boundaries were prepared for gauge stations. Catchment boundaries were adopted from GSIM (The Global Streamflow Indices and Metadata Archive)[56] according to the station coordinates for most sites. Following the catchment delineation procedure[57], missing catchment boundaries were supplemented using information from our datasets for other sites. We then used ArcGIS Map to extract specific environmental factors.

The availability of catchment boundaries for each station enables association of environmental variables to each gauge by extracting them from corresponding global-scale gridded products. As summarized in Supplementary Table 6, 600 gauges of catchment-scale metadata were derived from six global data products chosen to represent natural and anthropogenic categories of catchment characteristics, with lithology[58] and Köppen–Geiger climate[59, 60] taken as natural factors. Land cover, irrigation, dam, and population were considered as anthropogenic environmental factors.

For lithology in GLiM (Global Lithological Map), we reclassified the original 15 lithological subclasses and obtained four new lithological sub-classes (sedimentary, volcanic (igneous) rocks, metamorphic rocks, and ice). The Köppen–Geiger climate classification system, which comprised 31 climate classes each described by a three-letter code, was reclassified into arid, temperate, tropical, cold, and polar types. In the same way, GLCNMO (Global Land Cover by National Mapping Organization)[61] was reclassified into vegetation, urban, agriculture, and bare land. Moreover, we selected irrigation as a percentage of total grid cell area[62], DOR (degree of regulation) from GRanD (Global Reservoir and Dam Database)[63] and discharge in our datasets related to dams, and population density from GPWv4 (Gridded Population of the World version 4)[64] as representative quantitative anthropogenic factors. We then plotted the latitudinal distribution of typical environmental factors (carbonate sedimentary rocks, acid volcanic rock, arid climate classification, temperate climate classification, global irrigation area, and urban land cover classification) as the percentage ratio of the specific factor area at a given latitude to its worldwide area in Fig. 5c and d, and Supplementary Fig. 7.

Finally, we extracted sub-classified factors of each global product for all available catchments using ArcGIS Map. Then we summarized the proportional information on the various environmental factors for 600 river stations. These metadata (comprising percentages of different classes of catchment characteristics, and representing the characteristics of the upstream catchment for each streamflow gauge) were calculated from the gridded data masked.

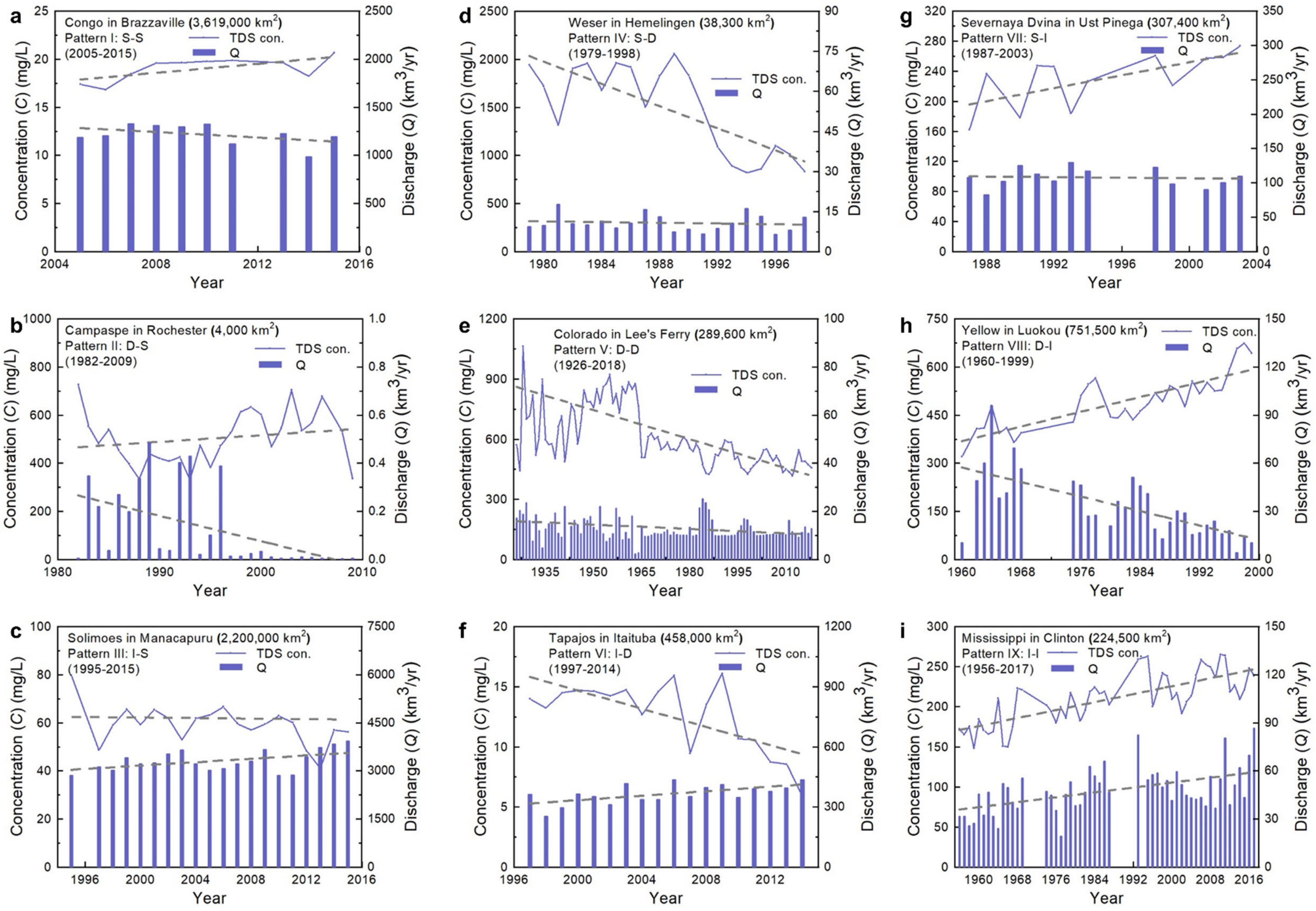

Supplementary Fig. 1 Representative large rivers for nine [*Q*, *C*] co-patterns showing discharge (*Q*, histograms), total dissolved

solids (TDS) concentrations (*C*, solid line), and corresponding trends (grey dashed lines). In the nine patterns, S represents stable trend, I represents increasing trend, and D represents decreasing trend. (**a**) Congo River at Brazzaville, (**b**) Campaspe River at Rochester, (**c**) Solimoes River at Manacapuru, (**d**) Weser River at Hemelingen, (**e**) Colorado River at Lee's Ferry, (**f**) Tapajós River at Itaituba, (**g**) Severnaya Dvina River at Ust Pinega, (**h**) Yellow River at Luokou, and (**i**) Mississippi River at Clinton. All trends are significant at the 0.05 significance level, and the dashed lines are the regression lines.

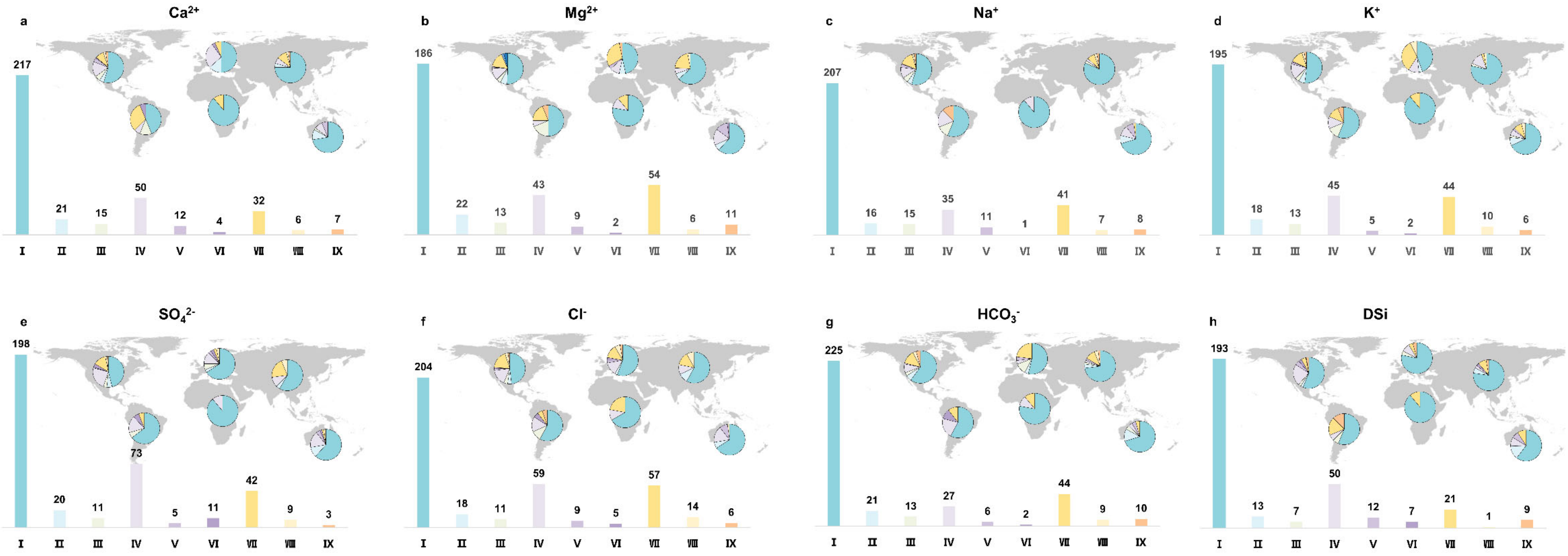

Supplementary Fig. 2 Nine [*Q, C*] co-varying patterns for discharge (*Q*) and dissolved solids concentrations (*C*), and their global distributions. **a~h** represent Ca^{2+}, Mg^{2+}, Na^{+}, K^{+}, SO_4^{2-}, Cl^{-}, HCO_3^{-}, and dissolved silica (DSi). Histograms show the distribution of nine patterns in each continent and the number of river stations exhibiting each pattern in the world's large rivers. All trends are significant at the 0.05 significance level.

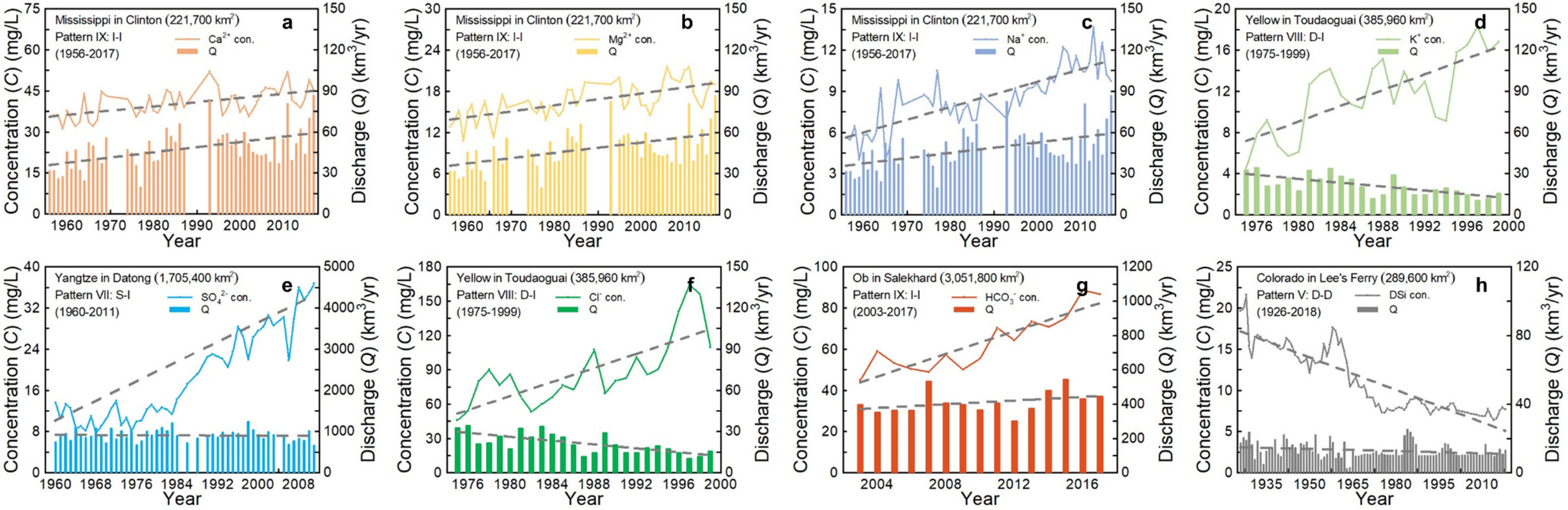

Supplementary Fig. 3 Representative large rivers for nine [*Q*, *C*] co-varying patterns showing discharge (*Q*, histograms), dissolved ion concentrations (*C*, solid line), and corresponding trends (grey dashed lines). In the nine patterns, S represents stable trend, I represents increasing trend, and D represents decreasing trend. (**a**) Ca^{2+}: Clinton at Mississippi River, (**b**) Mg^{2+}: Clinton at Mississippi River, (**c**) Na^{+}: Clinton at Mississippi River, (**d**) K^{+}: Toudaoguai at Yellow River, (**e**) SO_4^{2-}: Datong at Yangtze River, (**f**) Cl^{-}: Toudaoguai at Yellow River, (**g**) HCO_3^{-}: Salekhard at Ob' River, (**h**) dissolved silica (DSi): Lee's Ferry at Colorado River. All trends are significant at the 0.05 significance level.

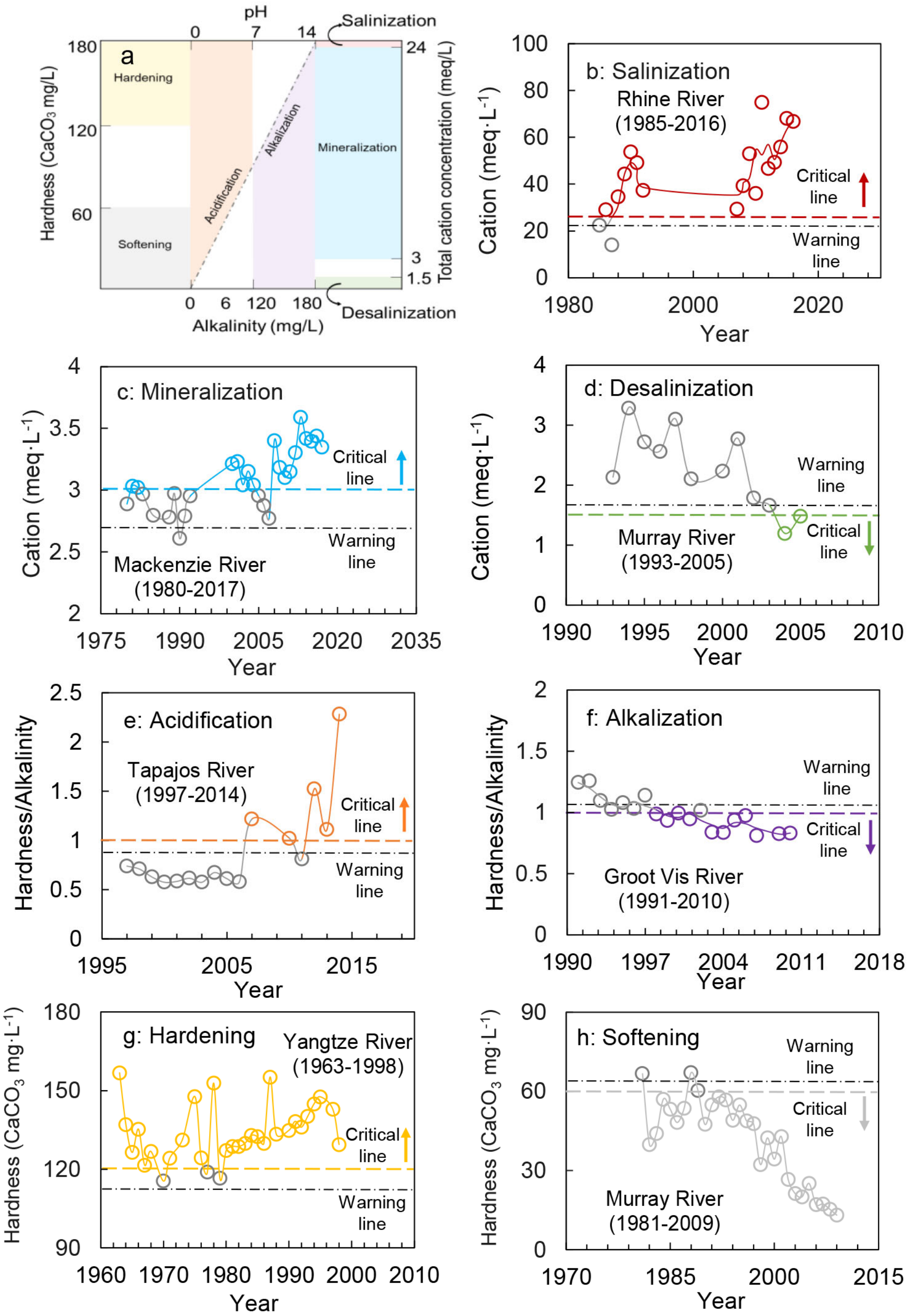

Supplementary Fig. 4 Warning signs and thresholds of solute metrics for identification of solute-induced river syndromes. a illustrates the thresholds of solute metrics used to identify solute-induced river syndromes. **b~h** present seven river syndromes associated with temporal variation in solute concentration. The critical line indicates the threshold used to identify a solute-induced river syndrome. The warning line represents the status when a specific solute metric reaches 90% of one of the

corresponding thresholds (e.g. Σ^+ (Ca^{2+}+Mg^{2+}+Na^++K^+) at 21.6 meq/L, 2.7 meq/L, and 1.7 meq/L for salinization, mineralization, and desalinization; hardness/alkalinity at 0.9 and 1.1 for acidification and alkalization; and hardness ($CaCO_3$) at 108 mg/L and 66 mg/L for hardening and softening).

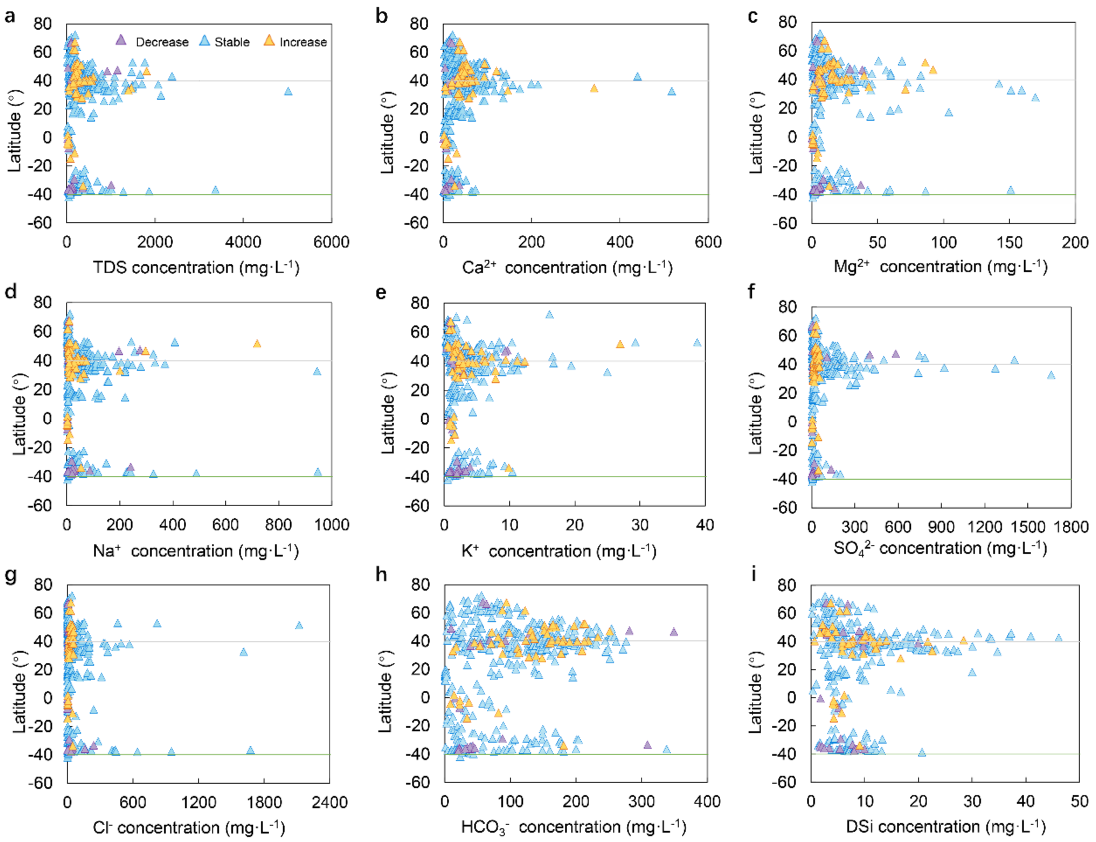

Supplementary Fig. 5 Latitudinal distribution of mean annual solute concentrations. **a**, Total dissolved solids (TDS). **b**, Ca^{2+}. **c**, Mg^{2+}. **d**, Na^{+}. **e**, K^{+}. **f**, SO_4^{2-}. **g**, Cl^{-}. **h**, HCO_3^{-}. **i**, Dissolved silica (DSi). Purple, blue, and yellow triangles respectively represent rivers with decreasing, stable, and increasing trends in solutes.

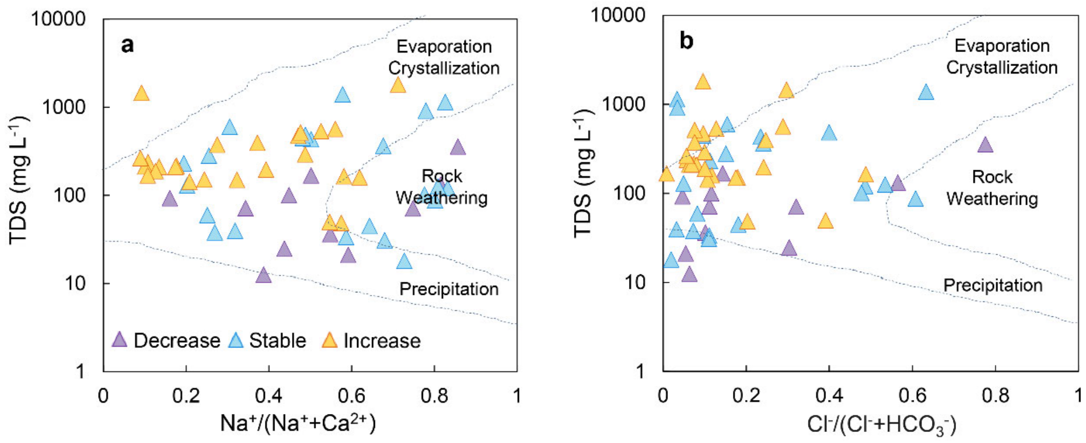

Supplementary Fig. 6 Gibbs model for stations with solute-induced river syndromes with varying trends in total dissolved solids (TDS) concentrations in three typical latitudinal belts. Purple, blue, and yellow triangles respectively represent gauge stations with decreasing, stable, and increasing TDS trends.

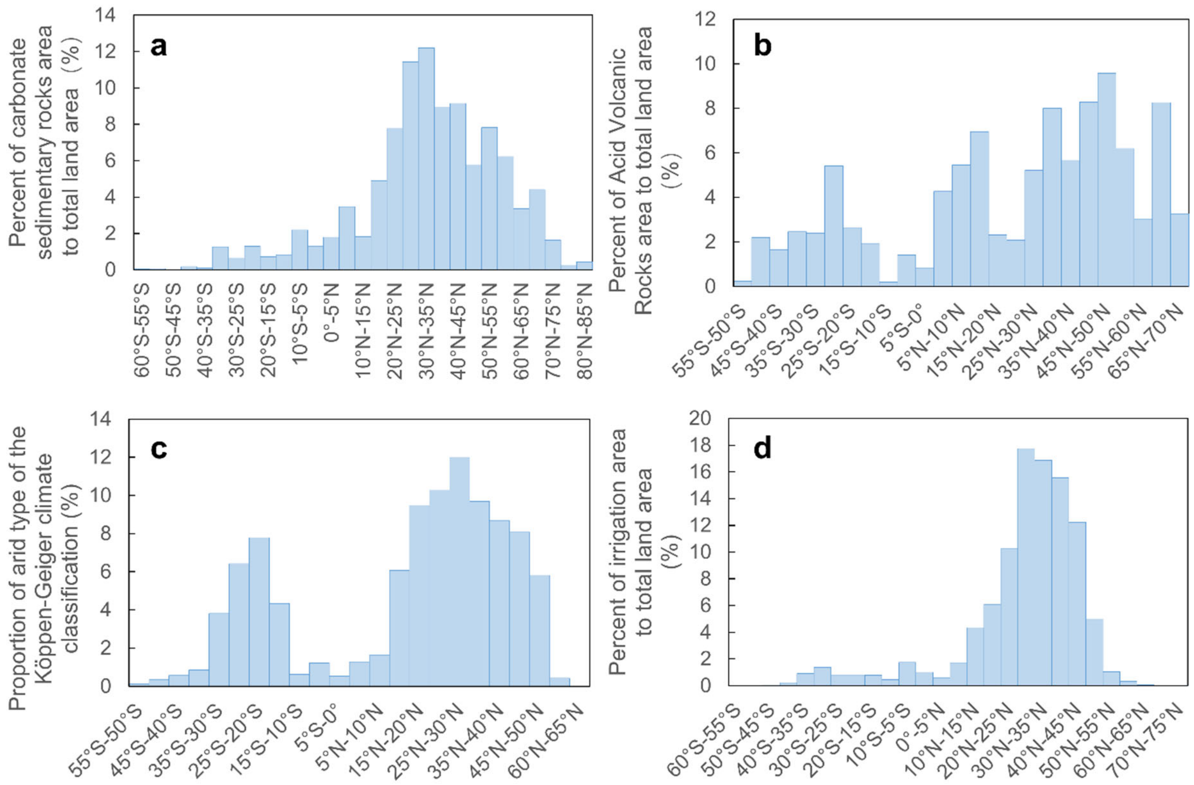

Supplementary Fig. 7 Latitudinal distribution of typical environmental factors. **a**~**d** show latitudinal distributions of carbonate sedimentary rock, acid volcanic (igneous) rocks, arid climate classification, and irrigation area.

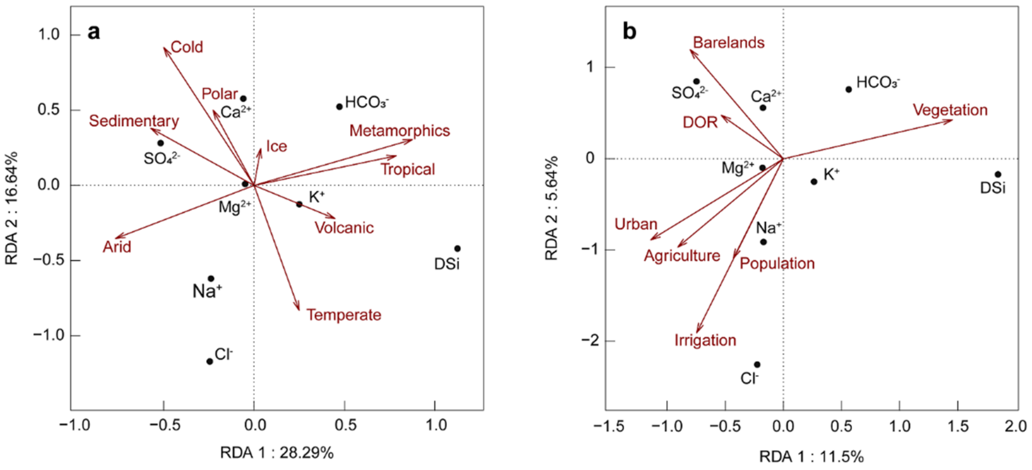

Supplementary Fig. 8 Redundancy analysis (RDA) results for dissolved solids and environmental factors in the world's large rivers. a, for natural factors (reclassification for Köppen climate and lithology) in 68 sites with solute-induced river syndromes. **b**, for anthropogenic factors (irrigation, population, degree of regulation (DOR), and detailed reclassification of land cover) in 68 sites with solute-induced river syndromes. DSi represents dissolved silica.

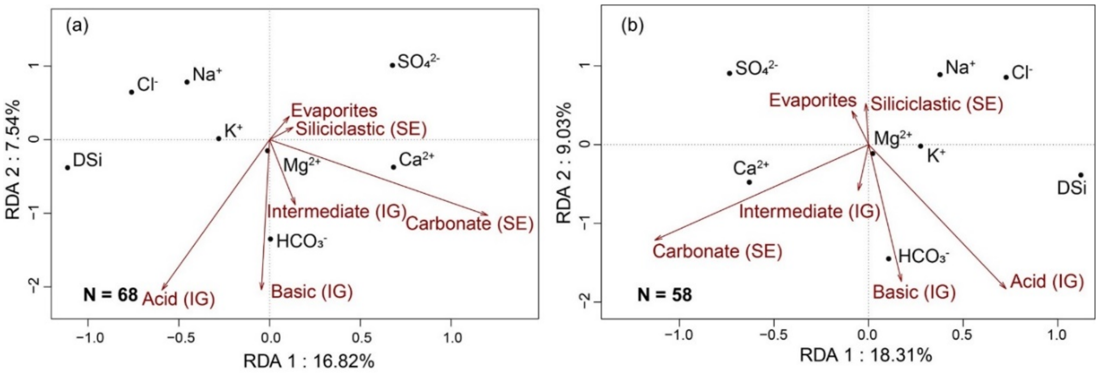

Supplementary Fig. 9 Redundancy analysis (RDA) for dissolved solids in the world's large rivers for natural factors (sub-classification of sedimentary and igneous (volcanic) rocks). a, in 68 sites with solute-induced river syndromes. **b**, in 58 sites with solute-induced river syndromes in critical latitudinal belts. IG and SE represent igneous and sedimentary rocks. DSi represents dissolved silica.

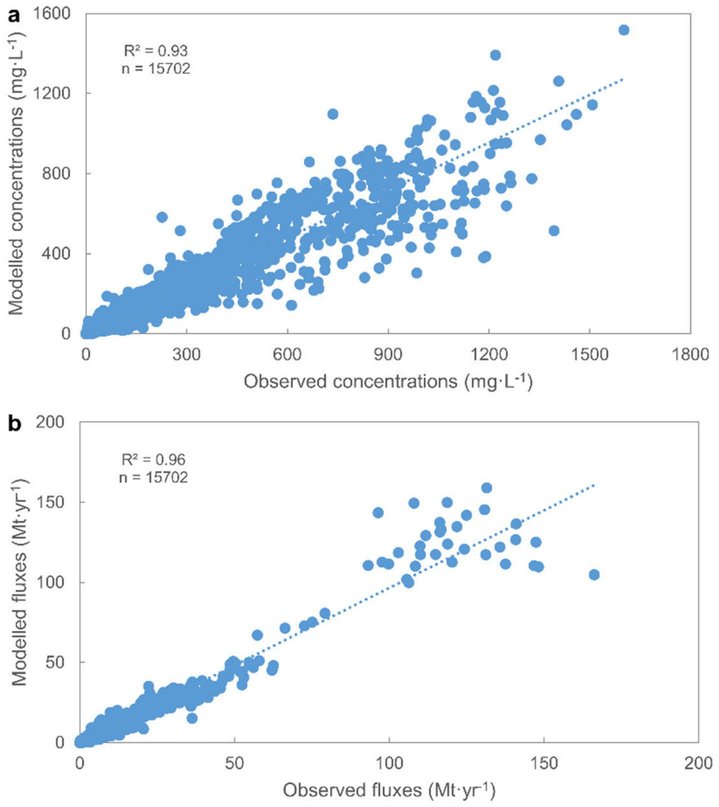

Supplementary Fig. 10 Comparison between observed and modeled dissolved solid concentrations (a) and fluxes (b), and linear regressions (dashed lines).

Supplementary Table 1 Summary of underlying natural and human causes of water quality in total dissolved solids (TDS) trends for nine [Q, C_{TDS}] co-varying patterns of discharge (Q) and TDS concentrations (C_{TDS})

Pattern	Representative gauge station in world's large river	Causes for [Q, C_{TDS}] trends
Pattern I	Congo River at Brazzaville	The Brazzaville station in the Congo River of Africa had relatively stable Q and C_{TDS} (Pattern I) from 2005-2015, as shown in Supplementary Fig. 1a. This is reasonable because the river is located in a geologically old and highly degraded shield, and its basin comprises 50% tropical rain forest and has experienced negligible changes in precipitation over several decades[65, 66].
Pattern II	Campaspe River at Rochester	The large decrease of annual discharge in the Campaspe River (Supplementary Fig. 1b), affecting the whole of southern Australia[67], likely resulted from lower autumn and winter rainfall, fewer high rainfall years, and increased temperature[68]. However, effective salinity control in the Rochester Irrigation Area has helped maintain stable C_{TDS} over time[69].
Pattern III	Solimoes River at Manacapuru	The Solimoes River, the largest tributary of the Amazon River, experienced stable TDS and increasing river discharge between 1995-2015 at the Manacapuru station (Pattern III, Supplementary Fig. 1c). This was due to increasing atmospheric water vapor import from the warming tropical Atlantic and intensified chemical weathering by higher precipitation, which compensated C_{TDS} dilution by the increased river discharge[70].
Pattern IV	Weser River at Hemelingen	The Weser River had a sharp decline of TDS and a stable river discharge trend between 1979 and1998 (Pattern IV, Supplementary Fig. 1d), due to reduced human interference (e.g., mining, agriculture, etc.) and effective conservation management[71, 72].
Pattern V	Colorado River at Lee's Ferry	The Colorado River at Lee's Ferry station exhibited decreasing Q and C_{TDS} between 1926 and 2018 (Pattern V, Supplementary Fig. 1e), which could be attributed to damming and conservation management for decreasing TDS[73], severe climate change[74], droughts[75] and increasing water withdrawal in the upper Colorado River Basin from 1995 to 2000 for decreasing river discharge[76].

Supplementary Table 1 Summary of underlying natural and human causes of water quality in total dissolved solids (TDS) trends for nine [Q, C_{TDS}] co-varying patterns of discharge (Q) and TDS concentrations (C_{TDS}) (*continued*)

Pattern	Representative gauge station in world's large river	Causes for [Q, C_{TDS}] trends
Pattern VI	Tapajós River at Itaituba	The Tapajós River, the fifth largest tributary of the Amazon River in the central north of Brazil[77], experienced decreasing C_{TDS} and increasing river discharge (Pattern VI, Supplementary Fig. 1f) between 1997 and 2014. Compared with the Amazonian Solimoes River, the discharge of the Tapajós River was ten times lower because of its smaller basin size. Limited TDS loading, river dilution[78] and basin effects[70] could have contributed to the decreasing trend in TDS concentration.
Pattern VII	Severnaya Dvina River at Ust Pinega	In the Arctic region, the Severnaya Dvina (S. Dvina) River at Ust Pinega station (Pattern VII, Supplementary Fig. 1g) exhibited no river discharge trend variations resulting from longer ice duration[79, 80]. The significant increase in TDS concentration was probably due to accelerated elemental cycling and export through increased plant productivity in a warming climate[81, 82].
Pattern VIII	Yellow River at Luokou	The discharge at the Luokou station in the lower reach of the Yellow River displayed a sharp decreasing trend, caused by decreasing precipitation, and increasing water withdrawal for intensified agricultural irrigation[83], whereas the increasing C_{TDS} trend is mainly attributed to the concentration effect of low river flow[65] and saline irrigation return waters[7] (Pattern VIII, Supplementary Fig. 1h).
Pattern IX	Mississippi River at Clinton	At Clinton Station, the Mississippi River showed increasing Q and C_{TDS} between 1956-2017 (Supplementary Fig. 1i, in agreement with Raymond et al.[84] who reported increasing river discharge and HCO_3^- concentrations, mainly due to agricultural practices (e.g., fertilizer use, liming, and irrigation). The present study corroborates earlier findings that continental US rivers have been experiencing freshwater salinization syndrome[85].

Supplementary Table 2 Estimated global fluxes of dissolved solids to the oceans

Discharge	Year	Area	TDS	Ca^{2+}	Mg^{2+}	Na^{+}	K^{+}	SO_4^{2-}	Cl^{-}	HCO_3^{-}	DSi	References
km^3/yr		Mkm^2					Mt/yr					
31400	1989	/	3600	500	124.8	138	46.8	294	117.15	1989	390	[86]
37400	1999	148.17	3618	500	126	195	48	202	217	1946	389	[87]
36000	2011	105	3800	/	/	/	/	/	360	/	330	[71]
39080	2019	114.7	6393	783	215	362	78	711	652	2421	363	Present study

Note: TDS represents total dissolved solids; DSi represents dissolved silica.

Supplementary Table 3 Summary of redundancy analysis (RDA) vector reports for dissolved solids concentrations and typical natural environmental factors at 68 sites with solute-induced river syndromes and 58 syndrome sites in the critical latitudinal belts (“***”, “**”, “*”, “.”, and “ ” represent correlations at significant levels of 0.001, 0.01, 0.05, 0.1, and 1 (2-tailed); r^2 indicates correlation coefficient square; α indicates significance. The symbols have the same meanings in the following tables)

Environmental factors	68 sites					58 sites				
	R1	R2	r^2	*p*	α	R1	R2	r^2	*p*	α
Tropical	0.98	0.20	0.23	0.001	***	-0.98	-0.18	0.25	0.001	***
Arid	-0.93	-0.37	0.25	0.001	***	0.93	0.36	0.26	0.001	***
Temperate	0.34	-0.94	0.28	0.001	***	-0.47	0.88	0.27	0.001	***
Cold	-0.53	0.85	0.41	0.001	***	0.61	-0.79	0.43	0.001	***
Polar	-0.46	0.89	0.11	0.024	*	0.51	-0.86	0.13	0.022	*
Sedimentary	-0.85	0.53	0.18	0.001	***	0.91	-0.42	0.17	0.01	**
Volcanic	0.90	-0.43	0.09	0.032	*	-0.90	0.43	0.12	0.038	*
Metamorphic	0.96	0.28	0.30	0.001	***	-0.94	-0.33	0.33	0.001	***
Ice	0.12	0.99	0.02	0.367		0.97	0.26	0.01	0.817	

Supplementary Table 4 Summary of redundancy analysis (RDA) vector reports for dissolved solids concentrations and typical anthropogenic environmental factors at 68 sites with solute-induced river syndromes and 58 syndrome sites in the critical latitudinal belts

Environmental factors	68 sites					58 sites				
	R1	R2	r^2	*p*	α	R1	R2	r^2	*p*	α
Population	-0.57	-0.82	0.04	0.300		0.95	0.32	0.03	0.508	
Vegetation	1.00	0.09	0.13	0.012	*	-1.00	0.03	0.14	0.016	*
Agriculture	-0.89	-0.46	0.07	0.086	.	1.00	-0.03	0.09	0.066	.
Bare land	-0.79	0.62	0.08	0.064	.	0.87	-0.50	0.11	0.052	.
Urban	-0.94	-0.33	0.09	0.042	*	1.00	0.05	0.10	0.072	.
Irrigation	-0.57	-0.82	0.12	0.028	*	0.76	0.64	0.13	0.019	*
DOR	-0.89	0.45	0.03	0.439		0.71	-0.71	0.06	0.193	

Note: DOR represents degree of regulation.

Supplementary Table 5 Summary of redundancy analysis (RDA) vector reports for dissolved solids concentrations and sub-classified rocks at 68 sites with solute-induced river syndromes and 58 syndrome sites in the critical latitudinal belts

Environmental factors	68 sites					58 sites				
	R1	R2	r^2	*p*	α	R1	R2	r^2	*p*	α
Acid (IG)	-0.78	-0.62	0.19	0.001	***	0.87	-0.49	0.23	0.002	**
Basic (IG)	0.08	-1.00	0.12	0.019	*	0.17	-0.99	0.10	0.050	*
Intermediate (IG)	0.64	-0.77	0.03	0.336		-0.52	-0.86	0.01	0.686	
Evaporites	0.84	0.54	0.01	0.860		0.14	0.99	0.01	0.868	
Siliciclastic (SE)	0.98	0.21	0.01	0.844		-0.56	0.83	0.01	0.816	
Carbonate (SE)	0.96	-0.26	0.47	0.001	***	-0.94	-0.35	0.51	0.001	***

Note: IG and SE represent igneous (volcanic) and sedimentary rocks, respectively.

Supplementary Table 6 Global data products used for extracting natural and anthropogenic factors driving water chemistry in the world's large rivers

Variables	Data sources	Spatial resolution	Reference period
Lithology	The Global Lithological Map v1.0 (GLiM) dataset (Hartmann and Moosdorf, 2012[58]) https://www.clisap.de/research/b:-climate-manifestations-and-impacts/ crg-chemistry-of-natural-aqueous-solutions/global-lithological-map/ (last access: 15 Dec. 2019)	0.5 arc deg × 0.5 arc deg	--
Climate type	World map of Köppen–Geiger climate classification system (Rubel and Kottek, 2010[59]) http://koeppen-geiger.vu-wien.ac.at (last access: 15 Dec. 2019)	5 arcmin × 5 arcmin	1951-2000
Land cover	Global Land Cover by National Mapping Organizations (GLCNMO) (Tateishi et al., 2011[61]) http://www.iscgm.org/ (last access: 15 Dec. 2019)	30 arcsec × 30 arcsec	2003
Irrigation	Global Map of Irrigation Areas version 5 (Siebert et al., 2019[62]) http://www.fao.org/aquastat/en/geospatial-information/ global-maps-irrigated-areas/latest-version (last access: 15 Dec. 2019)	5 arcmin × 5 arcmin	Around 2005
Dams	Global Reservoir and Dam (GRanD), version 1 (Lehner et al., 2011[63]) http://sedac.ciesin.columbia.edu/data/set/grand-v1-dams-rev01 (last access: 15 Dec. 2019)	6862 data points storage capacity of more than 0.1 km^3	--
Population	Gridded Population of the World (GPW) version 4 (CIESIN, 2016[64]) http://sedac.ciesin.columbia.edu/data/set/gpw-v4-population-count (last access: 15 Dec. 2019)	30 arcsec × 30 arcsec	2005

Supplementary Table 7 Data sources for concentrations of dissolved ions in world's large rivers

Data sources	Number of sites	Record years	Access time
Arctic Great Rivers Observatory (ARCTIC-GRO)	6	2003-2017	Sep. 2018
Australian databases (Provincial Survey of New South Wales, Waterwatch Victoria, and Environmental Protection Agency for South Australia)	64	1973-2008	Sep. 2019
Canada's National Water Data Archive (HYDAT)	37	1970-2017	Sep. 2018
Confederación Hidrográfica del Ebro	1	1987-2014	Sep. 2018
Datenportal der Flussgebietsgemeinschaft (FGG) Elbe	1	1980-2016	Sep. 2018
Flussgebietsgemeinschaft Weser (FGG Weser)	2	1979-2016	Sep. 2018
Global Environment Monitoring System for Water (GEMS)	312	1966-2016	Sep. 2018
Hydrological Yearbooks of the People's Republic of China	5	1964-2011	Sep. 2018
Rijkswaterstaat	4	1960-2016	Sep. 2018
the Observation Service SO HYBAM (HYBAM)	18	1994-2016	Sep. 2018
United States Geological Survey (USGS)	190	1915-2018	Sep. 2019
Literature sources	29	1958-2012	Sep. 2018

Supplementary Table 8 Data sources for runoff in world's large rivers

Data sources	Number of sites	Record years	Access time
Arctic Great Rivers Observatory (ARCTIC-GRO)	6	2003-2017	Sep. 2018
Australian databases (Provincial Survey of New South Wales, Waterwatch Victoria, and Environmental Protection Agency for South Australia)	58	1973-2011	Sep. 2019
Canada's National Water Data Archive (HYDAT)	36	1970-2016	Sep. 2018
Confederación Hidrográfica del Ebro	1	1987-2014	Sep. 2018
Datenportal der Flussgebietsgemeinschaft (FGG) Elbe	1	1980-2016	Sep. 2018
Equipe d'Administration de la Banque HYDRO (HYDRO)	2	1979-1993	Sep. 2018
Flussgebietsgemeinschaft Weser (FGG Weser)	2	1979-2015	Sep. 2018
PKU-IEE- WLRs-WS-NL2006 Database	417	1946-2018	Sep. 2019
Rijkswaterstaat	4	1961-2016	Sep. 2018
The Observation Service SO HYBAM (HYBAM)	18	1994-2016	Sep. 2018
United States Geological Survey (USGS)	127	1951-2018	Sep. 2019
Literature and online web sources	29	1960-2015	Sep. 2018

Supplementary Table 9 Numbers of stations with dissolved solids concentration (DS, *C*) and river runoff (*Q*) data of various record lengths

Record length (Years)	Ca^{2+}	Mg^{2+}	Na^{+}	K^{+}	SO_4^{2-}	Cl^{-}	HCO_3^{-}	DSi[b]	TDS[c]	*Q*
<5	65	63	78	75	72	75	82	52	91	104
[5, 10)[a]	62	50	63	66	66	76	67	64	89	83
[10, 20)	147	132	139	130	141	149	152	114	131	151
[20, 30)	90	91	77	78	94	91	92	74	85	96
[30, 40)	40	41	38	38	41	47	41	37	42	43
[40, 50)	27	28	27	26	30	24	21	21	28	30
≥50	28	26	25	25	31	36	18	23	29	39
Sum	459	431	447	438	475	498	473	385	495	546

[a] Right parenthesis indicates that record length is not inclusive.
[b] Dissolved silica.
[c] Total dissolved solids.

Supplementary References

1. Chapman, D. V., World Health Organization. Water quality assessments: a guide to the use of biota, sediments and water in environmental monitoring (CRC Press, Cambridge, 1996).
2. Li, S. Y. & Bush, R. T. Changing fluxes of carbon and other solutes from the Mekong River. *Sci. Rep.* **5**, 1-16 (2015).
3. Müller, B., Berg, M., Pernet-Coudrier, B., Qi, W. & Liu, H. The geochemistry of the Yangtze River: Seasonality of concentrations and temporal trends of chemical loads. *Global Biogeochem. Cycles* **26**, 1-14 (2012).
4. Chen, J. S., Wang, F. Y., Xia, X. H. & Zhang, L. T. Major element chemistry of the Changjiang (Yangtze River). *Chem. Geol.* **187**, 231-255 (2002).
5. Dai, Z. J., Du, J. Z., Zhang, X. L., Su, N. & Li, J. F. Variation of Riverine Material Loads and Environmental Consequences on the Changjiang (Yangtze) Estuary in Recent Decades (1955-2008). *Environ. Sci. Technol.* **45**, 223-227 (2011).
6. Chen, J. S., et al. Spatial and temporal analysis of water chemistry records (1958-2000) in the Huanghe (Yellow River) basin. *Global Biogeochem. Cycles* **19**, 1-27 (2005).
7. Chen, J. S., He, D. W. & Cui, S. B. The response of river water quality and quantity to the development of irrigated agriculture in the last 4 decades in the Yellow River Basin, China. *Water Resour. Res.* **39**, 1-11 (2003).
8. Chen, J. S., Guan, W. R., Xia, X. H. & He, D. W. A probe into several problems of water-quality trends in the mainstream of Yangtze River from 1960's to 1980's.

Environmental Chemistry **1**, 8-13 (1998). in Chinese.

9. Xie, C. J., Gao, Q. Z., Tao, Z., Liu, L. H. & Li, S. C. Chemical weathering and CO_2 consumption in the Dongjiang River Basin. *Acta Scientiae Circumstantiae* **8**, 2123-2133 (2013). in Chinese.
10. Li, D. The study on the hydro-chemical characteristics and the flux to the sea about the Rivers in the East of China (East China Normal University, Shanghai, 2009). in Chinese.
11. Liu, X. C., Shen, H. T. & Huang, Q. H. Concentration variation and flux estimation of dissolved inorganic nutrient from the Changjiang River into its estuary. *Oceanologia et limnologia sinica* **3**, 332-340 (2002). in Chinese.
12. Shen, Z. L. A study on the effects of the Three Gorge Project on the distributions and changes of the nutrients in the Changjiang River estuary. *Oceanologia et limnologia sinica* **22**, 540-546 (1991). in Chinese.
13. Wang, J. C., Bian, J. J. & Chen, X. G. Analysis on changing tendency of water quality in trunk stream of Yangtze River Since 2000. *HubeiWater Power* **2**, 1-3 (2009). in Chinese.
14. Xia, X. Q., et al. Major ion chemistry in the Yangtze River. *Earth Sci. Front.* **15**, 194-202 (2008). in Chinese.
15. Xia, X. J., Xu, J., Feng, W. J. & Li, Q. The relationship between discharge to the sea and in Datong-Xuliujing of Yangtze River. *ZhongGuoShuiYun* **6**, 71-73 (2016). in Chinese.
16. Biggs, T., et al. Closing of the Krishna Basin: Irrigation, streamflow depletion and

macroscale hydrology (International Water Management Institute, Colombo, 2007).

17. Bouraoui, F. & Grizzetti, B. Long term change of nutrient concentrations of rivers discharging in European seas. *Sci. Total Environ.* **409**, 4899-4916 (2011).
18. Chetelat, B., et al. Geochemistry of the dissolved load of the Changjiang Basin rivers: Anthropogenic impacts and chemical weathering. *Geochim. Cosmochim. Acta* **72**, 4254-4277 (2008).
19. European Marine Observation and Data Network. Time series of annual water discharge. http://www.emodnet-arctic.eu/content/content.php?menu=19&htm=78# (2019).
20. Gao, Q. Z., et al. Chemical weathering and CO_2 consumption in the Xijiang River basin, South China. *Geomorphology* **106**, 324-332 (2009).
21. Gong, Y., et al. Seasonal Variation and Sources of Dissolved Nutrients in the Yellow River, China. *Int. J. Env. Res. Public Health* **12**, 9603-9622 (2015).
22. Jian, J., Webster, P. J. & Hoyos, C. D. Large-scale controls on Ganges and Brahmaputra river discharge on intraseasonal and seasonal time-scales. *Q. J. R. Meteorol. Soc.* **135**, 353-370 (2009).
23. Li, M. T., Xu, K. Q., Watanabe, M. & Chen, Z. Y. Long-term variations in dissolved silicate, nitrogen, and phosphorus flux from the Yangtze River into the East China Sea and impacts on estuarine ecosystem. *Estuar. Coast. Shelf Sci.* **71**, 3-12 (2007).
24. Li, S. Y., Lu, X. X. & Bush, R. T. Chemical weathering and CO_2 consumption in the Lower Mekong River. *Sci. Total Environ.* **472**, 162-177 (2014).
25. Li, S. Y. & Bush, R. T. Rising flux of nutrients (C, N, P and Si) in the lower Mekong

River. *J. Hydrol.* **530**, 447-461 (2015).

26. Lu, X. X., Li, S. Y., Kummu, M., Padawangi, R. & Wang, J. J. Observed changes in the water flow at Chiang Saen in the lower Mekong: Impacts of Chinese dams? *Quat. Int.* **336**, 145-157 (2014).
27. Negrel, P., Roy, S., Petelet-Giraud, E., Millot, R. & Brenot, A. Long-term fluxes of dissolved and suspended matter in the Ebro River Basin (Spain). *J. Hydrol.* **342**, 249-260 (2007).
28. Ollivier, P., Radakovitch, O. & Hamelin, B. Major and trace element partition and fluxes in the Rhône River. *Chem. Geol.* **285**, 15-31 (2011).
29. OSPAR Commission. Technical supplement 4 - Complementary graphic presentation of catchment data in 1990-2006: Assessment of riverine inputs and direct discharges of nutrients and selected hazardous substances to OSPAR maritime area in 1990-2006 (2019).
30. Ouyang, S., et al. Impact of Water Diversion of the South-to-North Water Diversion Middle Route Project on the Hydrological Characteristics at the Junction of Yangtze River and Poyang Lake (Destech Publications, Lancaster, 2016).
31. Ran, L. S., Lu, X. X., Sun, H. G., Han, J. T. & Yu, R. H. Chemical denudation in the Yellow River and its geomorphological implications. *Geomorphology* **231**, 83-93 (2015).
32. Singh, S. K., Sarin, M. M. & France L. C. Chemical erosion in the eastern Himalaya: Major ion composition of the Brahmaputra and δ13C of dissolved inorganic carbon. *Geochim. Cosmochim. Acta* **69**, 3573-3588 (2005).

33. Sun, H. G., Han, J. T., Li, D., Zhang, S. R. & Lu, X. X. Chemical weathering inferred from riverine water chemistry in the lower Xijiang basin, South China. *Sci. Total Environ.* **408**, 4749-4760 (2010).

34. Wortmann, M. Hydrological modelling of the Lena River using SWIM (Potsdam Institute for Climate Impact Research, Germany, 2014).

35. Yu, S., et al. Impacts of anthropogenic activities on weathering and carbon fluxes: a case study in the Xijiang River basin, southwest China. *Environ. Earth Sci.* **75**, 11 (2016).

36. Zhang, S. R., et al. Water chemistry of the Zhujiang (Pearl River): Natural processes and anthropogenic influences. *J. Geophys. Res.-Earth Surf.* **112**, 17 (2007).

37. Zhulidov, A., et al. Long-term dynamics of water-borne nitrogen, phosphorus and suspended solids in the lower Don River basin (Russian Federation). *J. Water Clim. Chang.* **2**, 201-211 (2011).

38. Xia, X. H. Water quality (major ions) evolution of the Yangtze river system from 1960's to 1990's (Peking University, Beijing, 1998). in Chinese.

39. Grubbs, F. E. Sample criteria for testing outlying observations. *Ann. Math. Stat.* **21**, 27-58 (1950).

40. Howell, D. C. Statistical methods in human sciences (Wadsworth, New York, 1998).

41. Wang, J. Contribution of Upstream Tributaries and Returned Irrigation Groundwater to Chemical Weathering in the Yellow River Basin (Ocean University of China, Qingdao, 2014). in Chinese.

42. Jacobson, A. D. & Blum, J. D. Relationship between mechanical erosion and atmospheric CO_2 consumption in the New Zealand Southern Alps. *Geology* **31**, 865-868 (2003).

43. Runkel, R. L., Crawford, C. G. & Cohn, T. A. Load Estimator (LOADEST): A FORTRAN program for estimating constituent loads in streams and rivers (U. S. Geological, Virginia, 2004).

44. Burnham, K. P. & Anderson, D. R. Model Selection and Multi- Model Inference: a Practical Information-Theoretic Approach (Springer-Verlag, New York, 2002).

45. Meybeck, M., Durr, H. H. & Vörösmarty, C. J. Global coastal segmentation and its river catchment contributors: A new look at land-ocean linkage. *Global Biogeochem. Cycles* **20**, 1-15 (2006).

46. Durr, H. H., Meybeck, M., Hartmann, J., Laruelle, G. G. & Roubeix, V. Global spatial distribution of natural riverine silica inputs to the coastal zone. *Biogeosciences* **8**, 597-620 (2011).

47. Fekete, B. M., Vörösmarty, C. J. & Grabs, W. High-resolution fields of global runoff combining observed river discharge and simulated water balances. *Global Biogeochem. Cycles* **16**, 1-11 (2002).

48. Meybeck, M. & Ragu, A. River discharges to the oceans: an assessment of suspended solids, major ions and nutrients (United Nations Environment Programme, Paris, 1995).

49. Mann, H. B. Nonparametric test against trend. *Econometrica* **13**, 245-259 (1945).

50. Kendall, M. Rank correlation measures (Charles Griffin, London, 1976).

51. Yue, S., Pilon, P., Phinney, B. & Cavadias, G. The influence of autocorrelation on the ability to detect trend in hydrological series. *Hydrol. Processes* **16**, 1807-1829 (2002).

52. Meybeck, M. Global Occurrence of Major Elements in Rivers. *Treatise Geochem.* **5**, 207-223 (2003).

53. Drake, T. W., et al. Increasing Alkalinity Export from Large Russian Arctic Rivers. *Environ. Sci. Technol.* **52**, 8302-8308 (2018).

54. Global Environment Monitoring System for Water. Global Water Quality database. https://gemstat.org (2018).

55. McGowan, W. Water processing: residential, commercial, light-industrial 3rd edn (Water Quality Assiciation, Lisle, 2000).

56. Do, H. X., Gudmundsson, L., Leonard, M. & Westra, S. The Global Streamflow Indices and Metadata Archive (GSIM) - Part 1: The production of a daily streamflow archive and metadata. *Earth Syst. Sci. Data* **10**, 1-21 (2018).

57. Bai, R., Li, T., Huang, Y., Li, J. & Wang, G. An efficient and comprehensive method for drainage network extraction from DEM with billions of pixels using a size-balanced binary search tree. *Geomorphology* **238**, 56-67 (2015).

58. Hartmann, J. & Moosdorf, N. The new global lithological map database GLiM: A representation of rock properties at the Earth surface. *Geochem., Geophys., Geosyst.* **13**, 1-37 (2012).

59. Rubel, F. & Kottek, M. Observed and projected climate shifts 1901–2100 depicted by world maps of the Köppen-Geiger climate classification. *Meteorol. Z.* **19**, 135-

141 (2010).

60. Peel, M. C., Finlayson, B. L. & McMahon, T. A. Updated world map of the Köppen-Geiger climate classification. *Hydrol. Earth Syst. Sci.* **11**, 1633-1644 (2007).

61. Tateishi, R., et al. Production of global land cover data – GLCNMO. *Int. J. Digit. Earth* **4**, 22-49 (2011).

62. Siebert, S., Henrich, V., Frenken, K. & Burke., J. Global Map of Irrigation Areas version 5. http://www.fao.org/aquastat/en/geospatial-information/global-maps-irrigated-areas/latest-version (2019).

63. Lehner, B., et al. High-resolution mapping of the world's reservoirs and dams for sustainable river-flow management. *Front. Ecol. Environ.* **9**, 494-502 (2011).

64. Center for International Earth Science Information Network. Documentation for the Gridded Population of the World, Version 4 (GPWv4). http://dx.doi.org/10.7927/H4D50JX4 (2016).

65. Laraque, A., et al. A comparison of the suspended and dissolved matter dynamics of two large inter-tropical rivers draining into the Atlantic Ocean: the Congo and the Orinoco. *Hydrol. Processes* **27**, 2153-2170 (2013).

66. Samba, G., Nganga, D. & Mpounza, M. Rainfall and temperature variations over Congo-Brazzaville between 1950 and 1998. *Theor. Appl. Climatol.* **91**, 85-97 (2008).

67. Mosley, L. M., et al. The impact of extreme low flows on the water quality of the Lower Murray River and Lakes (South Australia). *Water Resour. Manage.* **26**,

3923-3946 (2012).

68. Potter, N. J. & Chiew, F. H. S. An investigation into changes in climate characteristics causing the recent very low runoff in the southern Murray-Darling Basin using rainfall-runoff models. *Water Resour. Res.* **47**, 1-12 (2011).
69. Chiew, F. H. S. & McMahon, T. A. Groundwater recharge from rainfall and irrigation in the Campaspe River Basin. *Aust. J. Soil Res.* **29**, 651-670 (1991).
70. Chen, J. S. River water quality in China (Science Press, Beijing, 2006). in Chinese.
71. Milliman, J. D. & Farnsworth, K. L. River discharge to the coastal ocean: a global synthesis (Cambridge University Press, New York, 2011).
72. Barles, S. Urban metabolism and river systems: an historical perspective - Paris and the Seine, 1790-1970. *Hydrol. Earth Syst. Sci.* **11**, 1757-1769 (2007).
73. Morford, S. L. Salinity in the Colorado River Basin. https://watershed.ucdavis.edu/education/classes/files/content/page/6%20Morford-Colorado_Basin_Salinity.pdf (2014).
74. McCabe, G. J., Wolock, D. M., Pederson, G. T., Woodhouse, C. A. & McAfee, S. Evidence that Recent Warming is Reducing Upper Colorado River Flows. *Earth Interact.* **21**, 1-14 (2017).
75. Udall, B. & Overpeck, J. The twenty-first century Colorado River hot drought and implications for the future. *Water Resour. Res.* **53**, 2404-2418 (2017).
76. Maupin, M. A., Ivahnenko, T. I. & Bruce, B. Scientific Investigations Report: Estimates of water use and trends in the Colorado River Basin, Southwestern United States, 1985–2010 (U. S. Geological Survey, Reston, 2018).

77. Farinosi, F., et al. Future Climate and Land Use Change Impacts on River Flows in the Tapajós Basin in the Brazilian Amazon. *Earth's Future* **7**, 993-1017 (2019).

78. Pantoja, N. G. P., et al. Quality of the Solimoes River water for domestic use by the riverine community situated in Manacapuru-Amazonas-Brazil. *Environ. Sci. Pollut. Res.* **23**, 11395-11404 (2016).

79. Shiklomanov, A. I. & Lammers, R. B. River ice responses to a warming Arctic-recent evidence from Russian rivers. *Environ. Res. Lett.* **9**, 1-9 (2014).

80. Smith, L. C. Trends in Russian Arctic river-ice formation and breakup, 1917 to 1994. *Phys. Geogr.* **21**, 46-56 (2000).

81. Pokrovsky, O. S., et al. Biogeochemistry of carbon, major and trace elements in watersheds of northern Eurasia drained to the Arctic Ocean: The change of fluxes, sources and mechanisms under the climate warming prospective. *C. R. Geosci.* **344**, 663-677 (2012).

82. Viers, J., et al. Seasonal and spatial variability of elemental concentrations in boreal forest larch foliage of Central Siberia on continuous permafrost. *Biogeochemistry* **113**, 435-449 (2013).

83. Miao, C. Y., Ni, J. R., Borthwick, A. G. L. & Yang, L. A preliminary estimate of human and natural contributions to the changes in water discharge and sediment load in the Yellow River. *Global Planet. Change* **76**, 196-205 (2011).

84. Zhang, Y. K. & Schilling, K. E. Increasing streamflow and baseflow in Mississippi River since the 1940 s: Effect of land use change. *J. Hydrol.* **324**, 412-422 (2006).

85. Kaushal, S. S., et al. Freshwater salinization syndrome on a continental scale. *Proc.*

Natl. Acad. Sci. U. S. A. **115**, E574-E583 (2018).

86. Sarin, M. M., Krishnaswami, S., Dilli, K., Somayajulu, B. L. K. & Moore, W. S. Major ion chemistry of the Ganga-Brahmaputra river system: Weathering processes and fluxes to the Bay of Bengal. *Geochim. Cosmochim. Acta* **53**, 997-1009 (1989).
87. Galy, A. & France L. C. Weathering processes in the Ganges-Brahmaputra basin and the riverine alkalinity budget. *Chem. Geol.* **159**, 31-60 (1999).
