# Supplementary material for: Global syndromes induced by changes in solutes of the world’s large rivers
Source: Nat Commun. 2021 Oct 12;12:5940. doi: 10.1038/s41467-021-26231-w (PMC8511150; doi:10.1038/s41467-021-26231-w)
Supplement: Supplementary file 3 — Reporting Summary [file 41467_2021_26231_MOESM3_ESM.pdf]

nature portfolio

Corresponding author(s): Jinren Ni

Last updated by author(s): Sep 8, 2021

Reporting Summary

Nature Portfolio wishes to improve the reproducibility of the work that we publish. This form provides structure for consistency and transparency in reporting. For further information on Nature Portfolio policies, see our Editorial Policies and the Editorial Policy Checklist.

Statistics

For all statistical analyses, confirm that the following items are present in the figure legend, table legend, main text, or Methods section.

n/a	Confirmed	
☐	☒	The exact sample size (*n*) for each experimental group/condition, given as a discrete number and unit of measurement
☒	☐	A statement on whether measurements were taken from distinct samples or whether the same sample was measured repeatedly
☐	☒	The statistical test(s) used AND whether they are one- or two-sided<br>*Only common tests should be described solely by name; describe more complex techniques in the Methods section.*
☐	☒	A description of all covariates tested
☐	☒	A description of any assumptions or corrections, such as tests of normality and adjustment for multiple comparisons
☐	☒	A full description of the statistical parameters including central tendency (e.g. means) or other basic estimates (e.g. regression coefficient) AND variation (e.g. standard deviation) or associated estimates of uncertainty (e.g. confidence intervals)
☐	☒	For null hypothesis testing, the test statistic (e.g. *F*, *t*, *r*) with confidence intervals, effect sizes, degrees of freedom and *P* value noted<br>*Give P values as exact values whenever suitable.*
☒	☐	For Bayesian analysis, information on the choice of priors and Markov chain Monte Carlo settings
☒	☐	For hierarchical and complex designs, identification of the appropriate level for tests and full reporting of outcomes
☒	☐	Estimates of effect sizes (e.g. Cohen's *d*, Pearson's *r*), indicating how they were calculated

Our web collection on statistics for biologists contains articles on many of the points above.

Software and code

Policy information about availability of computer code

Data collection	No software was used.
Data analysis	LOAD ESTimator model, R package and ArcGIS Map were used to analyse the data in this study.

For manuscripts utilizing custom algorithms or software that are central to the research but not yet described in published literature, software must be made available to editors and reviewers. We strongly encourage code deposition in a community repository (e.g. GitHub). See the Nature Portfolio guidelines for submitting code & software for further information.

Data

Policy information about availability of data

All manuscripts must include a data availability statement. This statement should provide the following information, where applicable:

- Accession codes, unique identifiers, or web links for publicly available datasets
- A description of any restrictions on data availability
- For clinical datasets or third party data, please ensure that the statement adheres to our policy

Data sources for concentrations of dissolved ions and runoff in world's large rivers are available within the paper and its supplementary information file. Data on solutes concentrations (C), annual runoff (Q), co-varying trend in Q and C, and the identified solute-induced river syndrome if applicable, at each river station have been deposited on figshare [https://doi.org/10.6084/m9.figshare.14910399]. The source data underlying Figs. 1–5 and Supplementary Figs. 1–10 are provided with this paper.

Field-specific reporting

Please select the one below that is the best fit for your research. If you are not sure, read the appropriate sections before making your selection.

☐ Life sciences ☐ Behavioural & social sciences ☒ Ecological, evolutionary & environmental sciences

For a reference copy of the document with all sections, see nature.com/documents/nr-reporting-summary-flat.pdf

Ecological, evolutionary & environmental sciences study design

All studies must disclose on these points even when the disclosure is negative.

Study description	This study investigated seven solute-induced river syndromes (salinization, mineralization, desalinization, acidification, alkalization, hardening, and softening) associated with global trends in major solutes (Ca2+, Mg2+, Na+, K+, SO42−, Cl−, HCO3−) and dissolved silica in the world's large rivers (basin areas ≥ 1,000 km2).
Research sample	A comprehensive dataset from 600 gauge stations in 149 large rivers (basin areas ≥ 1,000 km2)
Sampling strategy	According to the available time series data of annual runoff (Q) and concentrations (C) of solutes, 600 monitoring stations located in 149 rivers (basin areas ≥ 1,000 km2) were selected across 6 continents. The 149 rivers collectively drain 46 million km2 of watersheds. Solute-induced river syndromes, identified according to certain thresholds, are characterized by specific symptoms, impacts, spatial distributions, and representative rivers. Seven solute-induced river syndromes have been observed in the world's large rivers including salinization, mineralization, desalinization, acidification, alkalization, hardening, and softening, all of which are associated with trends in major solutes.
Data collection	All data used in this study were collected from publicly available databases, open literature and websites.
Timing and spatial scale	Our global database includes data from 600 stations in 149 rivers located across six continents. The time scale of data collected in this study ranges from 1915 to 2018. To our knowledge, this database is the most comprehensive to date in terms of spatial and temporal coverage of dissolved solids.
Data exclusions	To control data quality, we removed outliers in the database. First, we tested whether the time series data for any of dissolved solids at any given station followed a normal distribution. If so, then outliers were identified using the Grubbs' test using the R package 'outliers'. If not, any data points beyond three standard deviations (SD) of the mean were considered outliers. We removed 1,273 outliers using the Grubbs' test and 615 outliers using the three SD method. The percentage number of removed outliers was 2.3% of the total data points.
Reproducibility	All attempts to repeat the research were successful.
Randomization	Based on the unique dataset of major solutes (Ca2+, Mg2+, Na+, K+, SO42−, Cl−, HCO3-) and dissolved silica derived from 600 gauge stations in 149 rivers, nine co-varying trends in annual runoff (stable, increasing, decreasing) and solute concentration (stable, increasing, decreasing) were revealed in global rivers. Moreover, we proposed a framework of notable solute-induced river syndromes (salinization, mineralization, desalinization, acidification, alkalization, hardening, and softening) which could be identified using thresholds of solute concentrations and associated trends in the world's large rivers.
Blinding	This is not an experiment-based study.

Did the study involve field work? ☐ Yes ☒ No

Reporting for specific materials, systems and methods

We require information from authors about some types of materials, experimental systems and methods used in many studies. Here, indicate whether each material, system or method listed is relevant to your study. If you are not sure if a list item applies to your research, read the appropriate section before selecting a response.

Materials & experimental systems

n/a	Involved in the study	
☒	☐	Antibodies
☒	☐	Eukaryotic cell lines
☒	☐	Palaeontology and archaeology
☒	☐	Animals and other organisms
☒	☐	Human research participants
☒	☐	Clinical data
☒	☐	Dual use research of concern

Methods

n/a	Involved in the study	
☒	☐	ChIP-seq
☒	☐	Flow cytometry
☒	☐	MRI-based neuroimaging
